# Diagnostic Overshadowing in High-Functioning Autism: Mirtazapine, Buspirone, and Modified Cognitive Behavioral Therapy (CBT) as Treatment Options

**DOI:** 10.7759/cureus.39446

**Published:** 2023-05-24

**Authors:** Nihit Gupta, Mayank Gupta

**Affiliations:** 1 Psychiatry, Dayton Children's Hospital, Dayton, USA; 2 Psychiatry and Behavioral Sciences, Southwood Psychiatric Hospital, Pittsburgh, USA

**Keywords:** differential diagnosis, buspirone, high functioning autism, child and adolescent psychiatry, autism spectrum disorder (asd)

## Abstract

Diagnostic overshadowing (DO) is identified as a contributor to the missed diagnosis of individuals with an autism spectrum disorder (ASD). It has been used predominantly in the scientific literature and clinical settings to describe a phenomenon where a person's symptoms and behaviors are attributed solely to their primary diagnosis, rather than being recognized due to co-occurring conditions. DO is seen across many developmental disorders; however, females with autism may have a more difficult time getting diagnosed than males with autism because traditional diagnostic criteria for autism are often based on research that has primarily focused on males with autism. Likewise, the efficacy of approved psychopharmacological like selective serotonin reuptake inhibitors (SSRIs) and cognitive behavioral therapy (CBT) in individuals with ASD is not well established. Amidst these challenges, it’s imperative to underscore the need for screening these disorders and provide informed evidence-based treatment alternatives for shared decision-making. Mirtazapine has low but promising findings, though modified CBT has superior empirical support in the treatment of co-occurring conditions associated with ASD.

## Introduction

Advances in the knowledge of autism spectrum disorder (ASD) genetics are likely to help us better understand the variabilities in phenotypes including high-functioning ASD [1]. In 2013, Asperger's syndrome (AS) was removed from the Diagnostic and Statistical Manual of Mental Disorders, 5th Edition (DSM-5), where it was merged into the broader category of ASD [2]. The Centers for Disease Control and Prevention (CDC) estimates that one in 36 (eight-year-olds) has ASD, considerably higher in recent surveys [3]. Its prevalence can vary and is contingent on the population studied, the validity of the measurement tools, and the diagnostic criteria used. The prevalence of a family history and genetic regions have also been linked to ASD although it’s a highly heterogenous and complex disorder that is influenced by environmental factors and gene-environment interactions [4].

Recent research indicates that the term ASD-related diagnostic overshadowing (DO) is frequently responsible for a lack of accurate clinical diagnoses of ASD. There is a tendency to overlook the ASD symptoms due to the more prominent symptoms of co-occurring mental health conditions or neurodevelopmental disorders [5-6]. ASD may be misdiagnosed if the focus is on behavioral symptoms of ASD, resulting in a delay in diagnosis for individuals with atypical or milder symptoms. Due to the lack of knowledge about the emerging evidence and phenotypic variability, there is a probability to overlook or dismiss the possibility of ASD in certain individuals, particularly women and those of different races or ethnicities than the stereotype of ASD [7].

To address these issues around the timely diagnosis and treatment of ASD, it is imperative to appraise the possibility of DO in individuals with ASD who have other cooccurring conditions. ASD symptoms can be overshadowed by symptoms of other conditions such as attention deficit hyperactivity disorder (ADHD), anxiety, depression, or intellectual disability [8]. It is possible that communication and social impairments in ASD could present as a social anxiety disorder [9] or restricted and repetitive behaviors and interest (RRBI), which could be mistaken for symptoms of obsessive-compulsive disorder (OCD) [10]. It is essential for professionals to be aware of these possibilities and to conduct a comprehensive evaluation. In individuals with ASD, treatment options like selective serotonin reuptake inhibitors (SSRIs) may not be as effective at treating depression and anxiety as recommended in typically developing youths [11] and may experience more behavioral activation side effects of SSRIs such as agitation and insomnia [12]. In contrast, mirtazapine is an atypical antidepressant that does not fit into traditional antidepressant categories such as tricyclic antidepressants and SSRIs. Mirtazapine is unique among antidepressants because it works on multiple neurotransmitters and has sedative properties that can help with insomnia and anxiety.

In these cases, we highlight atypical clinical presentations in individuals with ASD, issues related to DO, and modifications in the strategies for treatment.

## Case presentation

Case 1

A 15-year-old male adolescent was referred to the outpatient clinic by his pediatrician for a psychiatric evaluation. According to the referral letter, he has been struggling with low mood, school refusal, and conflicts with family members for a period of the last four months. The pediatrician has known him since birth, has followed him monthly since his mental health symptoms started, and initiated sertraline 25 mg daily for a week, which was increased to 50 mg once a day. After initiating the medication, the symptoms got worse, and the medication was subsequently stopped. His medical workup was negative, was also started weekly psychotherapy with limited improvement. During his psychiatric assessment, it was evident that he had narrow interests, which include technology and video games. He was an honor-roll student with advanced knowledge of Greek history and self-taught Japanese language from YouTube. He admitted having difficulties with reciprocal relationships with peers, was often the victim of bullying, and preferred solitary activities. He reported difficulties with peers due to his rigidity around the rules of games they played together. He also had a mild speech delay and was preoccupied with Legos and reptiles growing up; his parents reported sensory problems related to sensitivity to loud noises, haircuts, taking showers, and dislike toward tight clothing.

He also reported that he preferred to read books, listen to music, and interact with his online friends. There was no evidence of any substance use or trauma history. On mental status examination, he had poor eye contact and was dressed casually, in loose clothes. He had monotone speech with difficulties in pragmatics, reported low mood and anxiety, and had a flat affect. Based on his history, symptoms, and degree of clinical impairment, the diagnosis of ASD was established. Both the patient and his family were provided extensive psychoeducation and agreed to initiate a low dose of mirtazapine 15 mg for his depressive symptoms. At the two weeks follow-up, the patient had reported a 40% reduction in the affective symptoms. His family also had a meeting with the school principal to address bullying and modified cognitive behavioral therapy sessions were initiated. After six to eight weeks of treatment, there were significant clinical improvements and remission of depressive illness, and the school also initiated an emotional support individualized education program (IEP) plan to meet his needs in the classroom.

Case 2

A 15-year-old female, with a long history of multiple, inpatient hospital stays for suicidal ideations and self-injury behavior, was referred to the outpatient clinic after stepping down from an inpatient hospital stay. Hospitalizations mostly resulted from transitioning back to school, social difficulties, and the inability to maintain friends.

Her mental health problems began at age 11, but she was unable to identify any triggers. She had cognitive behavioral therapy at age 12 but with limited benefits. The pediatrician started her on fluoxetine 10 mg daily, which caused increased irritability so she was switched to sertraline 25 mg. She reported suicidal thoughts within two weeks of starting sertraline and was hospitalized for the first time at 12 years of age. She has been taking psychotropics for the past three years, including fluoxetine 10 mg daily, sertraline 25 mg daily, clonidine 0.1 mg daily at bedtime, bupropion 150 mg extended release, and hydroxyzine 25 mg up to three times a day. Aripiprazole 2 mg was given to her during one of the hospitalizations to treat her mood instability, without improvement and causing weight gain. As a result, she was switched to quetiapine 50 mg daily, which made her more irritable. Pharmacogenomic testing was done given the multiple failed trials. Following the results, she was started on desvenlafaxine 50 mg ER daily along with mirtazapine 7.5 mg daily at bedtime for sleep. She reported partial benefit in her depression and anxiety symptoms with this combination and denied any side effects. Despite this, she was readmitted to the hospital due to a crisis in her social life and was started on escitalopram, a dose titrated up to 10 mg daily. Medical workups have been negative and she has been in therapy for three years with four different therapists. Currently, she receives weekly therapy.

After completing the partial hospitalization program, she stopped taking escitalopram because it did not help. She had been seeing her current provider for about four months now but is not interested in going back on any medications because she didn't find them helpful. She has been struggling with depression and anxiety lately and hasn't attended school for three weeks. She has recently started online schooling and feels better because she doesn't have to interact with anyone. One day, the psychiatrist decided to coordinate care with her therapist. They discussed her intense interest in anime and Pokémon. Autism spectrum disorder was suspected due to her struggles with social cues. She was referred for Autism Diagnostic Observation Schedule-Second Edition (ADOS-2) and other neuropsychological testing but tested negative. However, both providers felt she has impairment due to social and communication difficulties and has a very restricted and intense interest, including no interest in going to school. Hence, she was clinically diagnosed with an autism spectrum disorder. 

During one of the appointments, the patient stated that desvenlafaxine and mirtazapine have been the only two medications that have helped in the past, and she wished to return to mirtazapine as a sleep aid. She was started on mirtazapine 7.5 mg daily for a week and then 15 mg daily at bedtime. On the four-week and eight-week follow-up, she reported improvement in her symptoms of depression and anxiety. Her PHQ-9 (9-question patient health questionnaire (PHQ-9) score was 20/27 at baseline, 12/27 at four weeks, and 10/27 at eight weeks. Similarly, her general anxiety disorder-7 (GAD-7) scores were 18/21 at baseline, 12/21 at four weeks, and 9/21 at eight weeks.

Case 3

A 16-year-old male adolescent presented to a rural outpatient clinic for mood management after being referred by his pediatrician. He had been irritable and agitated. He also used cannabis regularly. Moreover, he reported being depressed and anxious for the past two years. Since he and his parents had not been getting along, he had been living with his grandmother.

His grandmother had been concerned about his lack of friends and impulsivity. Additionally, she worried about his marijuana use, but he reported that it is the only medication that has ever helped him. The medications that he had been prescribed include fluoxetine, sertraline, escitalopram, and bupropion. The patient reported being more irritable and aggressive on fluoxetine, escitalopram, and sertraline. Although bupropion made him feel more "up and about," he didn't feel it helped with his depression or anxiety. Methylphenidate ER 18 mg daily had also been prescribed to him, which he initially found beneficial, but did not find additional benefits when the dosage was increased to 72 mg. His family found him to be more irritable on 72 mg.

Upon examination, the patient appeared to be a tall, smart individual with a narrow interest in chemistry and finance. Despite his difficulties maintaining friends, he was able to maintain friendships only with kids who smoked marijuana with him. His interactions were different, such as being more concrete and logical. Inflexibility caused his girlfriend to break up with him in a previous relationship. Loud noises caused him sensory difficulties. His speech was not a problem, but he maintained poor eye contact and interjected the writer during the interaction, feeling irritable and impulsive.

After discussing the possibility of autism spectrum disorder with the family, we established a diagnosis. He was encouraged to maintain strict sobriety from any substances, including marijuana, and was closely monitored, including referral for counseling for substance abuse. Considering his previous adverse experiences, the patient requested a medication that does not cause any side effects. Therefore, buspirone was chosen at a dose of 10 mg twice daily and then increased to 15 mg twice daily. A one-month follow-up revealed improvement in the patient's overall anxiety symptoms and depression. PHQ-9 was 21/27 to 9/27 and GAD-7 was 15/21 to 6/21. His family also reported that he felt better on buspirone with no worsening of mood.

## Discussion

Given there is a significant increase in the burden of undiagnosed and untreated ASD, many seeking treatments present unique challenges for clinicians working with limited resources and training about the emerging evidence. Although there are many advances in knowledge and empirical research, it’s not yet translated into a meaningful difference in real-world clinical settings. We, therefore, focus on how symptoms/traits of ASD could present in clinical settings, often overshadowed due to its resemblance with other cooccurring conditions based on common office-based clinical presentations described in the cases above.

The American Academy of Pediatrics recommends screening for ASD at 18 and 24 months as part of their well-child visits, and during the routine developmental screening, providers may ask parents or caregivers about the child's communication, behaviors, and social interactions [13]. The Modified Checklist for Autism in Toddlers (M-CHAT), The Ages and Stages Questionnaires (ASQ), and The Communication and Symbolic Behavior Scales Developmental Profile (CSBS DP) are a few commonly used screening instruments. However, it is reported that these instruments have variable validity in different settings, which attributes to false negatives for those who have symptoms of ASD [14]. This is one of the reasons associated with missed and delayed diagnoses. In clinical samples, ASD is underrepresented among the youths referred to mental health services and is considered five times higher than in the community [15]. On many occasions, overlapping and transdiagnostic symptoms are observed during the initial presentation, which may represent co-morbidities of conditions like anxiety disorders, depression, tics/Tourette’s syndrome, OCD, psychosis, and ADHD.

The literature suggests modifications when evaluating high-functioning ASD, (also known as Asperger's syndrome) since the narrow interests, often referred to as special interests, may present as intense and all-consuming interests but are often ego-syntonic for individuals with ASD, unlike compulsive behaviors in OCD, which are ego-dystonic [10]. In contrast, they can also be a source of frustration for the families and difficulty for those who do not understand or share their interests. Due to the deficits in theory of mind, understanding nonverbal cues, and abstract reasoning (literal-mindedness), these individuals may have difficulties understanding or expressing their interests; therefore, it is imperative to be respectful and non-judgmental when asking questions about narrow interests in ASD [16-18]. Similarly, it may present in individuals with gender dysphoria in non-conforming youths, mania, and other co-occurring disorders, clearly suggesting the need for reviewing ASD symptoms in every individual in psychiatrically referred populations [19,20].

The gender differences in ASD, including the presence of eye contact and social camouflaging, are newer emerging understandings about the phenotypic variability in females, requiring updated clinical knowledge to identify the subtle nuances of clinical presentations and impairments wherein. Social camouflaging is common for high-functioning ASD (HF-ASD) individuals and is more prevalent in females with ASD [21]. On many occasions, it’s thought to be a social anxiety disorder, however, a developmental understanding of social deficits may provide more granular information to be able to accurately establish a clinical diagnosis [9]. Likewise, early and deep interest with attention to detail in science, technology, reading books, Japanese culture, crime thrillers, horror movies, music, learning new languages, building Legos, collecting cars/trucks, animals (reptiles), astronomy, and forensics could provide cues requiring context for further inquiries and clinical correlations.

There are other subtle cues, which when raising clinical doubts must inspire further detailed and systemic inquiries. During the clinical assessment, a review of ASD symptoms with detailed information about narrow restrictive interests, sensory processing, social deficits, and sensitivity to psychotropics is likely to provide leads in establishing clinical symptomatology. It is also important to be aware of the attributional bias during the developmental history from parental interviews.

A few other scenarios include a lack of response to multimodal interventions with worsening of presenting symptoms, temporal link in the emergence of suicidal thoughts and behaviors two to three weeks after an increase in the dose of SSRI, positive history for developmental delays, speech delays, splinter skills, and the presence of impairing sensory symptoms that could be indicative of further psychiatric assessment [22].

Establishing a chronology of the onset of psychiatric symptoms (prepubertal and often before the age of six) from a reliable collateral historian could be a useful indicator for identifying neurodevelopmental disorders. In summary, based on the review of these cases, many individuals with ASD present in late adolescence, there is a possibility of false negative screens and/or negative ADOS-2 test, and they often present due to complaints related to co-occurring conditions. Therefore, it’s imperative to complete a comprehensive review of systems relevant to ASD and obtain detailed developmental history that may likely guide clinical diagnosis.

Besides ABA, there are no other evidence-based interventions that improve core symptoms like social and communication problems and restrictive/repetitive behaviors. Although, researchers have argued optimizing psychopharmacologic treatments for cooccurring conditions like SD, irritability, anxiety, etc. could indirectly be effective in reducing core symptoms of ASD [23]. Therefore, targeting these disorders with appropriate intervention remains critical to the overall improvement of ASD symptomatology and outcomes.

It's not uncommon for younger individuals with ASD to have behavioral activation with SSRI [11] and a temporal link with the initiation of SSRI medication and the development of these symptoms including suicidal thoughts within two to three weeks. The polymorphism of serotonin transporters and receptors and the late maturation of serotonin 1C receptors (inhibitory) are considered plausible reasons for these activations. The rapid increase in the level of serotonin, with the imbalance of excitatory and inhibitory serotonergic systems, and the possible differential metabolism status of the cytochrome P450 system could be related to these effects. The awareness of SSRI dose-related activation is also an essential cue for further evaluation of ASD symptomatology [12]. While low-dose SSRI in older adolescents is less likely associated with behavioral activation, overall SSRI’s role is limited given the poor risk-benefit profile [11]. These issues often result in limited psychopharmacological treatment options for common comorbid conditions like generalized anxiety and major depression.

In individuals with ASD, one of the most common co-occurring disorders is ADHD, which when treated with stimulants, does not have the linear dose response seen in typically developing youths [24]. There are reports of worsening symptoms with higher doses of stimulants in the lower IQ ASD individuals, therefore incorporating this emerging evidence to modify dose titration schedules or using non-stimulants or off-label options. These highlight some unique treatment-emergent cues and how clinicians may further make clinical inquiries.

It’s also common to have false-negative gold standard tests like ADOS-2, but it is evident from empirical research that these instruments are merely suggestive of ASD and the diagnosis of ASD is clinical and does not require further testing if criteria are met.

Amidst these critical issues related to diagnostic overshadowing and limited evidence-based treatments in the following section, we highlight a few alternative options that although have weak empirical evidence could be beneficial in select individuals. First, a limited number of studies of mirtazapine have been shown to reduce anxiety, depression, and insomnia in individuals with ASD. In a study of 36 children and adolescents with ASD, mirtazapine reduced anxiety, depression, and insomnia symptoms better than a placebo [25]. A second study of 20 adults with ASD found that mirtazapine reduced symptoms of anxiety, depression, and insomnia and improved social interactions [26].

Similarly, buspirone is a unique anti-anxiety that works as a partial 5HT1A agonist and is less likely to cause behavioral activation. It was found to be beneficial [27] for anxiety in young individuals with ASD at low doses. It is an alternative option with a favorable risk and adverse effects profile in ASD.

Modified CBT for ASD may utilize visual aids, such as pictures or diagrams, to help the individual comprehend the concepts discussed. It may also be beneficial for the therapist to use more concrete language and avoid figurative language. Additionally, the therapist may provide specific instructions and guidance since some individuals with ASD may struggle with traditional CBT's flexibility and open-ended nature [28]. It has been shown that modified CBT for ASD is effective in treating conditions like anxiety, depression, and OCD symptoms in individuals with ASD. In addition, modified CBT has been shown to improve social interactions, communication, and overall functioning in individuals with ASD.

Common conditions causing diagnostic overshadowing are summarized in Table 1.

**Table 1:** Summary of factors leading to diagnostic overshadowing in ASD OCD: Obsessive-Compulsive Disorder, ASD: Autism Spectrum Disorder, BPD: Borderline Personality Disorder, HF-ASD: High Functioning Autism Spectrum Disorder, ADHD: Attention Deficit Hyperactivity Disorder, SSRIs: Selective Serotonin Reuptake Inhibitors, NSSI: Non-Suicidal Self Injury, RRBI: Restricted and Repetitive Behaviors and Interest, GD: Gender Dysmorphia

Overshadowing	Empirical Evidence	Clinical Significance
HF-ASD (Undiagnosed)	Depression and Anxiety [6]	Among the HFA, especially females, it is common to remain undiagnosed and manifest only as depression and anxiety. There is strong evidence of an increased risk of suicide, and it is critical to implement universal screening for ASD among teens and adults, especially ones who are admitted to inpatient due to suicidality.
Limited social needs	Social Anxiety Disorder [9]	Limited social needs are often confused with Social Anxiety Disorder but can be differentiated based on the presence of avoidance and desire to socialize among individuals with Social Anxiety Disorder compared to ASD
Intense interests	OCD [10]	OCD is a common comorbidity associated with ASD and usually has worse outcomes. However, it can be differentiated given that OCD is ego-dystonic while RRBI/intense interests are ego-syntonic
Irritability	Bipolar Disorder [17]	Irritability is common in ASD and is multifactorial. SSRIs lack evidence of benefits in ASD and could lead to behavioral activation which is sometimes incorrectly diagnosed with Bipolar. Universal screening for ASD, especially among adolescents with a history of activation or irritability or suicidality secondary to SSRI trial may be beneficial.
Theory of mind deficits, literal-mindedness	Personality Disorders [18]	Borderline PD can be confused and misdiagnosed as ASD as they share symptoms like a lack of empathy and poor interpersonal skills. Additionally, the presence of BPD traits increased the risk of suicide and self-harm in ASD
Gender non-conforming youths	ASD [19,20]	Studies have found that individuals with ASD are more likely to be diagnosed with GD, both of which have overlapping symptoms like social impairment, lower empathy, and sensory sensitivities. Additionally, studies have also indicated that gender-diverse individuals are more likely to self-report autistic symptoms.
Hyperactivity	ADHD [24]	There is considerable genetic, neurobiological, and symptom (social interactions and executive functioning) overlap between ADHD and ASD, and can be difficult to differentiate. Stimulants are commonly prescribed, but the response is suboptimal in ASD with a predisposition to side effects. Moreover, stimulants may not follow a linear dose-response curve, and it is best to keep the dose low with frequent administrations during the day.
RRBI	NSSI [29]	NSSI is common in ASD and could be secondary to stress. NSSI can also be predictive of a future suicide attempt. On the other hand, it can also manifest as sensory-seeking behaviors and sometimes, can be totally independent of an individual’s depressive status.
Stereotypies	Tics [30]	Tics are commonly comorbid with ASD and can be difficult to differentiate from stereotypies. Stereotypies are usually fixed with prolonged duration compared to sudden onset tics. No medications have been found to be beneficial with stereotypies while tics usually respond to alpha-2 agonists.
Sensory issues	Insomnia [31]	Insomnia in ASD can sometimes be due to sensory issues which are common. It may be beneficial to screen the environment for sensory stimuli. Individuals with ASD may show a better response to long-acting Melatonin and are predisposed to side effects with other medications.

## Conclusions

A late diagnosis of ASD has many reasons including limited awareness and understanding of autism among healthcare professionals and the public, particularly those with HF-ASD or AS, who may have milder symptoms or be able to mask them more effectively, making them more difficult to diagnose.

The late diagnosis of autism can have negative consequences for the individual and their family, as it can limit their access to appropriate interventions and services that could help them reach their full potential. It's important for professionals to be aware of the possibility of a late diagnosis of autism and to consider the possibility of autism in individuals who have not been previously diagnosed, particularly those who have a history of developmental delays, social or communication challenges, and other neurodevelopmental disorders. Diagnostic overshadowing can delay the diagnosis of autism, particularly in individuals who may not fit the typical stereotype of ASD or who may have co-occurring conditions that mask their ASD symptoms. Therefore, screening these individuals seen in primary care and using evidence-based treatments to address their mental health symptoms is important.

## Appendices

**Table 2:** Key summary

Key Summary
ASD is overrepresented (up to five times more than community samples) in the psychiatrically referred child, adolescents, and transitional-age youths.
Screening for ASD symptoms is critical during a psychiatric evaluation, which includes a chronology of age of symptom onset, detailed developmental history, and ROS relevant to neurodevelopmental disorders.
It’s not uncommon for individuals with ASD to initially present with symptoms of ADHD, aggression/ irritability, SD, OCD, psychosis, social anxiety, etc. Therefore, it’s imperative to be aware of diagnostic overshadowing and cognizant of clinical cues which should inspire further inquiries into ASD symptoms and traits.
ASD remains a clinical diagnosis, but there are many caveats related to the validity of gold-standard tests like ADOS-2, and ADI which delay and may have false negative results.
The treatment of cooccurring conditions needs knowledge about the modifications in the class of psychopharmacologic treatments, dose-related side effects, activation syndrome, and the possible need for CYP 450 metabolism status. The treatment of medical comorbidities like GI symptoms may also yield improvement in other areas.
Although core symptoms have no evidence-based psychopharmacologic interventions, there are reports of improvement in symptoms of cooccurring disorders that may indirectly improve overall ASD symptomatology and outcomes.
Multimodal psychosocial interventions like modified CBT social skills training when available must be included for improved clinical outcomes.
The psychoeducation about the overshadowing related delays in the diagnosis, emerging evidence, and self-help materials has the potential for developing therapeutic alliance towards shared decision-making and treatment planning.
It’s also important to screen for other common psychiatric cooccurring disorders, and medical conditions like GI problems and refer to specialists when appropriate.
